# Evaluation of the radiomodifying effects of *Ginkgo biloba* in radioresistant triple negative breast cancer cells

**DOI:** 10.3389/fpubh.2026.1952136

**Published:** 2026-10-05

**Authors:** Sachin Gulati, Diana Seresova, Matus Durdik, Pavol Kosik, Lukas Jakl, Dominika Kochanova, Katarina Vigasova, Eva Markova, Igor Belyaev

**Affiliations:** Department of Radiobiology, Cancer Research Institute, Biomedical Research Center, Slovak Academy of Sciences, Bratislava, Slovakia

**Keywords:** apoptosis, cell viability, *Ginkgo biloba*, MDA-MB-231 cells, radiation response, reactive oxygen species (ROS), triple-negative breast cancer

## Abstract

Triple-negative breast cancer (TNBC) is an aggressive breast cancer subtype characterized by limited therapeutic options and intrinsic resistance to ionizing radiation. *Ginkgo biloba* (GB) possesses documented anticancer, antioxidant and pro-apoptotic properties; however, its potential role as a radiomodifying agent remains insufficiently investigated. The present study evaluated the effects of GB on the radiation response of the radioresistant MDA-MB-231 TNBC cell line. Cells were treated with increasing concentrations of GB (10–350 μg/mL) either alone or in combination with a clinically relevant single dose of 3 Gy delivered using 6 MV X-ray photons (3 Gy/min). Cell viability was evaluated by the MTT assay, while intracellular reactive oxygen species (ROS) generation and apoptosis were assessed by flow cytometry using CellROX and Annexin V/propidium iodide (PI) staining, respectively. Statistical evaluation was performed using two-way analysis of variance (ANOVA) to assess the effects of GB pretreatment, irradiation and their interaction. GB pretreatment produced a significant concentration-dependent reduction in cell viability, demonstrating intrinsic cytotoxic activity at higher concentrations. In contrast, neither 3 Gy irradiation alone nor the interaction between GB pretreatment and irradiation significantly affected cell viability. Similarly, no significant effects of GB, irradiation or their interaction were detected for intracellular ROS generation or the percentage of live cells determined by flow cytometry. These findings indicate that, under the present experimental conditions, GB did not significantly modify the response of MDA-MB-231 cells to clinically relevant irradiation. In conclusion, GB exhibits concentration-dependent cytotoxic activity toward MDA-MB-231 cells but no statistically significant radiomodifying effects of GB were detectable following a single 3 Gy exposure using the investigated short-term cellular endpoints.

## 1 Introduction

Breast cancer is the leading cause of cancer-related mortality among women and represents one of the most prevalent malignancies worldwide (1). It is a highly heterogeneous disease comprising several molecular subtypes that differ in their biological behavior, prognosis and response to therapy (2, 3). Among these, triple-negative breast cancer (TNBC) is characterized by the absence of estrogen receptor (ER), progesterone receptor (PR) and human epidermal growth factor receptor 2 (HER2) expression (4). TNBC accounts for approximately 15–20% of all diagnosed breast cancers and is associated with poor clinical outcomes, including rapid disease progression, early recurrence, and an increased risk of distant metastases (5). Because TNBC lacks established molecular therapeutic targets, treatment relies largely on chemotherapy and radiotherapy (6) although emerging nanotechnology-based immunomodulatory strategies are also under investigation (7). However, intrinsic radio-resistance remains a major obstacle to effective treatment (8). The MDA-MB-231 cell line is widely used as a model of radioresistant TNBC because of its aggressive phenotype, invasive behavior and relatively low sensitivity to ionizing radiation (9–12).

Radiotherapy exerts its antitumor effects primarily by inducing DNA damage through ionizing radiation (13). Although highly effective, its clinical application is limited by the balance between tumor control and radiation-induced normal tissue toxicity (14, 15). Consequently, considerable effort has been devoted to identifying radiomodifying agents that improve the therapeutic ratio of radiotherapy. Among the various strategies explored to improve the therapeutic efficacy of radiotherapy, increasing attention has been directed toward naturally derived compounds with potential radiomodifying properties.

Natural products have attracted increasing interest as potential radiomodifying agents because of their diverse biological activities and generally favorable safety profiles (16). Among these, *Ginkgo biloba* (GB) has been extensively investigated for its diverse pharmacological properties (17). Standardized *Ginkgo biloba* extracts such as EGb^®^ 761 are complex phytopharmaceutical preparations containing flavonoids glycosides and terpene lactones, principally ginkgolides and bilobalide, together with other minor constituents. These biologically active compounds have been associated with antioxidant, anti-inflammatory and cytoprotective effects (18, 19). In malignant cells, however, these constituents may also interfere with mitochondrial function, alter redox homeostasis and inhibit cellular proliferation (20, 21). Given the central involvement of oxidative stress, mitochondrial function and cell-death signaling in cellular responses to ionizing radiation, these properties provide a biological rationale for investigating whether GB can modify the radiation response of tumor cells. Several mechanisms have been proposed through which naturally derived compounds may modify radiation responses, including modulation of oxidative stress, DNA damage repair and apoptosis (22). In addition, regulation of epithelial-to-mesenchymal transition (EMT), a process associated with tumor invasion, metastasis and treatment resistance, has been reported for certain GB constituents (23). Nevertheless, it remains unclear whether these biological activities translate into measurable radiomodifying effects in radioresistant TNBC cells. To address this question, we investigated the effects of standardized GB extract (EGb^®^ 761) on the response of MDA-MB-231 cells to a clinically relevant single dose of 3 Gy ionizing radiation. Cell viability was evaluated using the MTT assay (24), while intracellular reactive oxygen species (ROS) generation (25) and Annexin V/propidium iodide flow cytometry (26) were employed to investigate oxidative stress and cell death. We hypothesized that GB would exert concentration-dependent cytotoxic effects and evaluated whether these effects translated into a measurable radiomodifying activity following clinically relevant irradiation.

## 2 Materials and methods

### 2.1 Chemicals

All chemicals were of analytical grade and were purchased from Sigma-Aldrich (St. Louis, MI, USA) and Merck (Darmstadt, Germany). The standardized *Ginkgo biloba* (GB) extract (EGb^®^ 761) was obtained from Dr. Willmar Schwabe Pharmaceuticals, Karlsruhe, Germany. EGb 761^®^ is a defined phyto-therapeutic dry extract standardized to contain 22.0–27.0% ginkgo flavone glycosides and 5.0–7.0% terpene lactones (including ginkgolides A, B, C, and bilobalide). It is a specific, non-homeopathic multi-component entity used as a research standard globally. The extract was dissolved in dimethyl sulfoxide (DMSO) to create a concentrated stock solution (40 mg/mL) and subsequently diluted in a culture medium to obtain final working concentrations. The final concentration of DMSO in all experimental groups was maintained below 0.1% (v/v) to avoid DMSO-induced toxicity. Dulbecco’s Modified Eagle’s Medium (DMEM), Fetal Bovine Serum (FBS), Penicillin–Streptomycin, Trypsin–EDTA, and Phosphate-Buffered Saline (PBS) were purchased from Sigma-Aldrich (St. Louis, MI, USA). Cell viability was determined using the MTT reagent [3-(4,5-dimethylthiazol-2-yl)-2,5-diphenyltetrazolium bromide] (Thermo Fisher Scientific, Waltham, MA, USA). Intracellular reactive oxygen species were measured using CellROX^®^ Green Reagent (Thermo Fisher Scientific, Waltham, MA, USA), and apoptosis was analyzed using the Annexin V-FITC/propidium iodide (PI) Apoptosis Detection Kit (Sigma-Aldrich, St. Louis, MO, USA).

### 2.2 Cell culture and characterization

The human triple-negative breast cancer (TNBC) parental cell line MDA-MB-231 (HTB-26™, ATCC, Manassas, VA, USA) was cultured in DMEM supplemented with 10% heat-inactivated FBS and 1% penicillin–streptomycin. The cultures were maintained at 37 °C in a humidified atmosphere containing 5% CO₂. Cells (P5-P8) were passaged upon reaching 80–90% confluence using 0.25% trypsin–EDTA. Only exponentially growing cells were used in all experiments.

### 2.3 Cell treatment and irradiation

Cells were treated with different concentrations of the standardized *Ginkgo biloba* (GB) extract EGb^®^ 761. Untreated cells served as controls for both the irradiated and non-irradiated experimental groups. Cells were pretreated with increasing concentrations of GB (0, 10, 50, 100, 200, 300, and 350 μg/mL) for 24 h before irradiation. Immediately before irradiation, the culture medium containing GB was removed and replaced with fresh complete medium. Irradiation was then performed in the absence of GB. *In vitro* irradiation was performed using a TrueBeam linear accelerator (Varian Medical Systems, Palo Alto, CA, USA) operating at a nominal beam energy of 6 MV X-ray photons and a dose rate of 3 Gy/min (Department of Radiation Oncology, St. Elisabeth Cancer Institute, Bratislava, Slovakia). A single dose of 3 Gy was selected because it represents a clinically relevant fractionation dose and permits evaluation of potential radiomodifying effects without inducing excessive radiation-mediated cytotoxicity. Cells (_˜_10,000 cells) cultured in 96-well plates were positioned within a custom configuration where the peripheral empty spaces of the plates were filled with pre-warmed water (~37 °C) to eliminate air cavities, ensuring electronic equilibrium and minimizing dose perturbation.

Samples were transported to and from the irradiation facility (approximately 20–30 min each way) under normothermic conditions (36 ± 1 °C) using a portable thermostatic system. Post-irradiation, the cell culture plates were immediately returned to a standard incubator (37 °C, 5% CO_2_) for subsequent functional analyses. Corresponding control samples were subjected to identical transportation and environmental conditions (sham-irradiation) for each experimental dose point. The technical parameters and dosimetric configuration of the irradiation setup were identical to those previously described (27). Intracellular ROS was assessed shortly after irradiation, whereas MTT and Annexin V/PI analyses were performed at 24, 48 and 72 h after irradiation.

### 2.4 Cell viability assay (MTT)

The cytotoxic and radiomodifying effects of GB were quantified using the MTT colorimetric assay. MDA-MB-231 cells were seeded in 96-well plates at a density of 10,000 cells per well. After cell attachment and treatment, 20 μL of MTT solution (5 mg/mL) was added to each well and the plates were incubated for 4 h at 37 °C. The resulting formazan crystals were dissolved in 150 μL of DMSO. The absorbance was measured at 570 nm using a microplate reader. Cell viability was expressed as a raw absorbance and viability trends were described qualitatively.

### 2.5 Measurement of intracellular reactive oxygen species (ROS)

Intracellular ROS levels were detected using the CellROX^®^ Green Reagent (Thermo Fisher Scientific, Waltham, MA, USA) according to the manufacturer’s instructions. Because the samples were transported back from the irradiation facility before staining, CellROX staining was initiated approximately 20–30 min after irradiation. Thus, ROS was assessed shortly after irradiation, rather than during irradiation itself. Tert-butyl hydroperoxide (TBHP, 200 μM) served as a positive control for ROS induction, cells were incubated at 37 °C for 45 min. Following GB pretreatment and 3 Gy irradiation, 2 μL of 2.5 mM CellROX^®^ Green solution was added directly to the cells in DMEM in each well and incubated for 45 min in a humidified CO_2_ incubator. Twenty minutes after CellROX addition, 3 μL of 7-AAD were added to stain dead cells. Samples were then incubated for another 25 min in CO_2_ incubator and subsequently harvested and analyzed by BD FACSCanto™ II flow cytometer. Data were analyzed via BD FACS Diva software. CellROX Green fluorescence was excited using the 488 nm blue laser and detected in the FITC channel (530/30-nm band pass filter), whereas 7-AAD fluorescence was excited using the 488 nm laser and detected using the appropriate far red fluorescence detector. Fluorescence compensation was established using the automatic compensation wizard implemented in FACSDiva software following acquisition of single-color stained and unstained control samples. The fluorescence intensity, reflecting the degree of ROS generation, was analyzed via flow cytometry (FACS) and expressed as mean fluorescence intensity (MFI). ROS fluorescence was quantified exclusively within the 7-AAD-negative (live-cell) population. GB concentrations (0, 75, 100, 200 and 300 μg/mL) used for the ROS analyses were selected based on the preceding MTT results to represent a range of concentrations spanning minimal to more pronounced effects on metabolic viability, while avoiding the highest concentration associated with marked cytotoxicity that could confound interpretation of flow-cytometric endpoints.

### 2.6 Apoptosis analysis by flow cytometry (FACS)

Apoptosis was evaluated by Annexin V-FITC/propidium iodide (PI) staining as previously described (28). Following treatment, MDA-MB-231 cells were detached using trypsin–EDTA, collected, centrifuged at 800 rpm for 5 min and resuspended in 100 μL of the Annexin V binding buffer (Roche, Basel, Switzerland). Cells were subsequently stained with Annexin V-FITC (Roche, Basel, Switzerland) and Propidium Iodide (PI) (Roche) according to the manufacturer’s instructions and incubated for 10 min in the dark at room temperature. After incubation, cells were washed with phosphate-buffered saline (PBS), centrifuged, resuspended in 200 μL of the Annexin V binding buffer, and analyzed using a BD FACS Canto II flow cytometer (BD Biosciences, San Jose, CA, USA). Annexin V-FITC and PI fluorescence were excited using a 488 nm laser and detected via 530/30 nm and 610/20 nm bandpass filters, respectively. A minimum of 10,000 events were acquired for each sample during flow cytometric analysis. Data acquisition and analysis were performed using BD FACSDiva software. Fluorescence compensation was established using single-stained and unstained control samples with the automatic compensation wizard implemented in the FACS Diva software. Cell populations were classified as live (Annexin V^−^/PI^−^), early apoptotic (Annexin V^+^/PI^−^), or late apoptotic/necrotic (LAN, Annexin V^+^/PI^+^). The percentages of each cell population were recorded for subsequent statistical analysis. As described for the ROS analysis, the GB concentrations used for apoptosis assessment were also selected based on the preceding MTT results.

### 2.7 Statistical analysis

All experiments were performed using three independent biological replicates. Data are presented as mean ± standard deviation (SD). Statistical analyses were performed using Statistica software (Dell Software, Round Rock, TX, USA). For comparisons among multiple treatment groups, one-way analysis of variance (ANOVA) followed by the least significant difference (LSD) *post-hoc* test was used where appropriate. To evaluate the independent effects of GB pretreatment, irradiation, and their interaction (GB × irradiation), two-way analysis of variance (ANOVA) was performed separately for each experimental endpoint and time point. For the MTT assay, GB concentration and irradiation were included as fixed factors for each incubation time (24, 48 and 72 h). Similar two-way ANOVA models were applied to the ROS and apoptosis datasets. Normality of model residuals was assessed using the Shapiro–Wilk test, and homogeneity of variance was evaluated using Levene’s test. The GB × irradiation interaction was used to determine whether GB exerted a radiomodifying effects. A *p*-value < 0.05 was considered statistically significant.

## 3 Results

### 3.1 Cell viability assay (MTT)

The MTT assay was performed to evaluate the cytotoxic effects of *Ginkgo biloba* (GB) extract and its potential influence on the response of MDA-MB-231 breast cancer cells to 3 Gy irradiation. Cell viability was assessed at 24, 48 and 72 h after treatment to determine concentration and time dependent effects of GB in both non-irradiated and irradiated cells. GB pretreatment induced a clear concentration-dependent reduction in cell viability at all three time points (Figure 1). Lower concentrations (10–100 μg/mL) produced only limited effects on cell viability, whereas higher concentrations (200–350 μg/mL) resulted in a marked reduction in viable cells. This concentration-dependent cytotoxic effect was consistently observed at 24, 48 and 72 h. No consistent time-dependent pattern was observed. Although minor differences were present among the three incubation periods, the overall response of MDA-MB-231 cells remained primarily dependent on GB concentration rather than treatment duration. Comparison of irradiated and non-irradiated cells showed only small numerical differences in cell viability at the corresponding GB concentrations. Although combined treatment with GB and irradiation tended to produce lower mean viability values than GB pretreatment alone at some concentrations, these differences were not statistically significant. To further evaluate whether GB modified the cellular response to irradiation, a two-way analysis of variance (ANOVA) was performed separately for each time point using GB concentration and irradiation as independent factors. GB concentration had a highly significant effect on cell viability (24 h: *F* (6, 28) = 48.120, *p* < 0.001; 48 h: *F* (6, 28) = 37.323, *p* < 0.001; 72 h: *F* (6, 28) = 26.917, *p* < 0.001). In contrast, irradiation alone did not significantly affect cell viability (24 h: *F* (1, 28) = 1.388, *p* = 0.553; 48 h: *F* (1, 28) = 0.275, *p* = 0.604; 72 h: *F* (1, 28) = 2.457, *p* = 0.128). Likewise, no significant interaction between GB concentration and irradiation was detected (24 h: *F* (6, 28) = 0.159, *p* = 0.986; 48 h: *F* (6, 28) = 0.024, *p* = 0.999; 72 h: *F* (6, 28) = 0.277, *p* = 0.943). Overall, these findings demonstrate that the reduction in cell viability was attributable to the intrinsic cytotoxic effects of GB. Under the present experimental conditions, 3 Gy irradiation did not significantly affect the viability of MDA-MB-231 cells, and GB did not significantly modify the cellular response to irradiation. Thus, no evidence of a radiomodifying effects of GB were detected based on the MTT assay.

**Figure 1:**
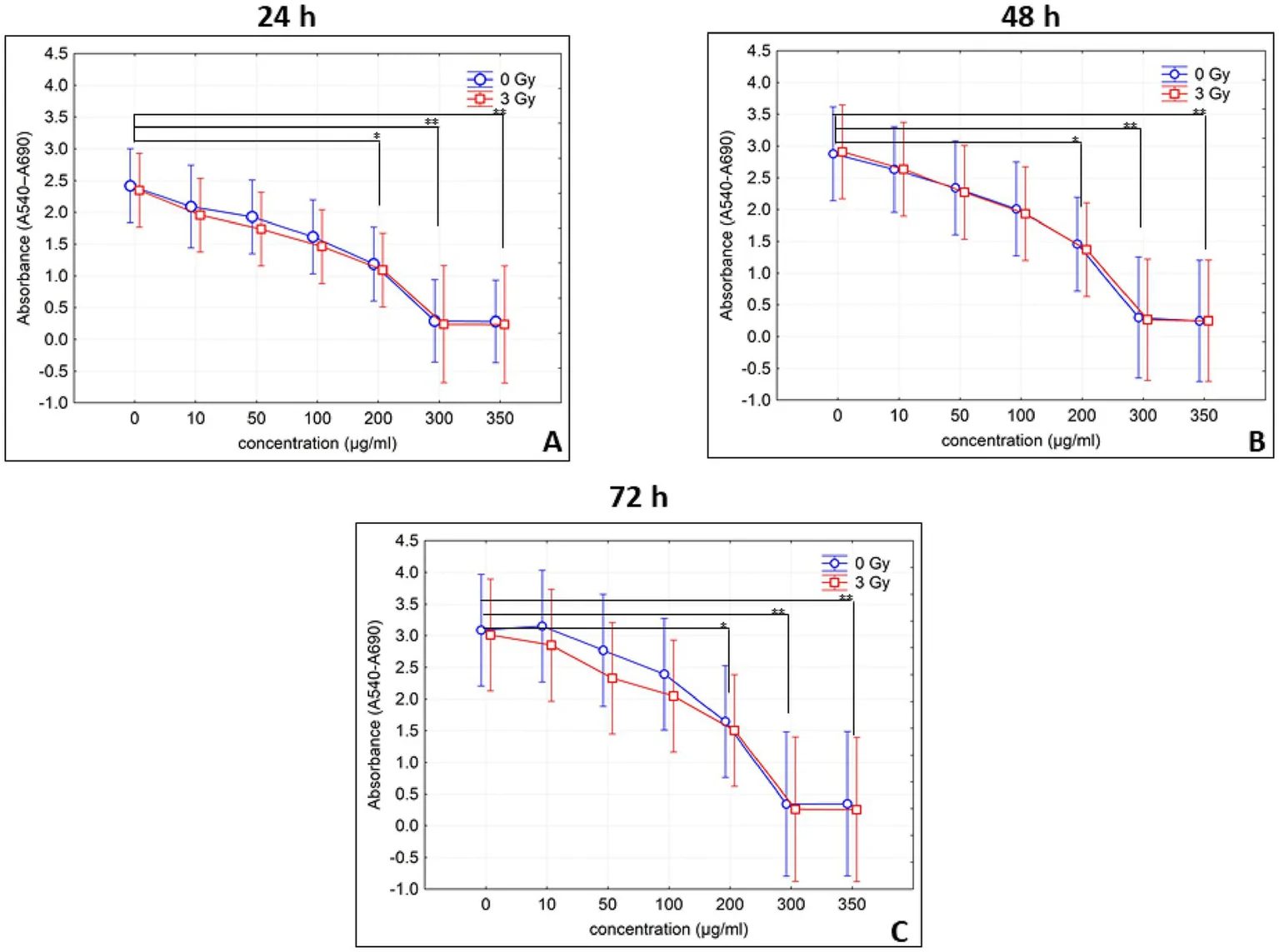
Concentration- and time dependent effects of *Ginkgo biloba* (GB) treatment, alone and in combination with 3 Gy irradiation, on the viability of MDA-MB-231 cells as determined by MTT assay. Cell viability was assessed at 24 h **(A)**, 48 h **(B)** and 72 h **(C)** following treatment with increasing concentrations of GB. Data are presented as mean ± SD from three independent biological experiments. Asterisks indicate significant differences compared with the corresponding untreated control (0 μg/mL GB): *p* < 0.05 (*) and *p* < 0.01 (**).

### 3.2 Measurement of intracellular reactive oxygen species (ROS) in live cells

Intracellular reactive oxygen species (ROS) generation in MDA-MB-231 cells was evaluated by flow cytometry using CellROX staining following treatment with selected concentrations of *Ginkgo biloba* (GB), chosen based on the MTT assay results, prior to exposure to 3 Gy irradiation. ROS levels and the percentage of live cells were assessed in both non-irradiated and irradiated groups to determine whether GB modified intracellular oxidative status or the cellular response to irradiation. Tert-butyl hydroperoxide (TBHP, 200 μM) served as a positive control for ROS induction. As expected, the positive control (TBHP) induced a marked increase in intracellular ROS levels, confirming the responsiveness of the assay (Figure 2A).

**Figure 2:**
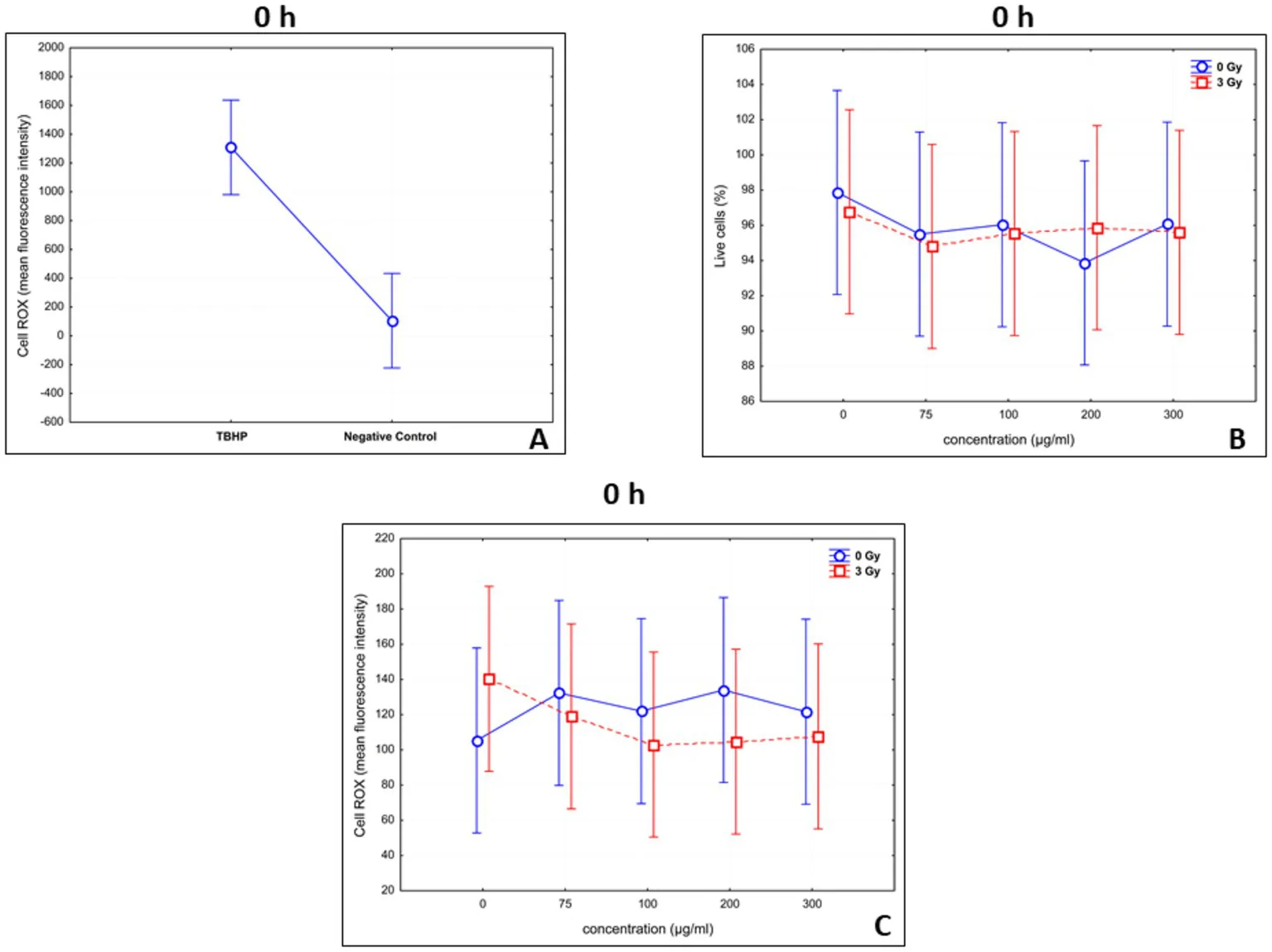
Effects of *Ginkgo biloba* (GB) treatment, alone and in combination with 3 Gy irradiation, on intracellular reactive oxygen species (ROS) generation in MDA-MB-231 cells, assessed by flow cytometry using CellROX^®^ Green staining. Cells treated with TBHP (positive control) and untreated cells (negative control) are shown in panel **(A)**. Cells were treated with increasing concentrations of GB (0–300 μg/mL), and the percentage of live cells **(B)** and intracellular ROS levels **(C)** were evaluated in non-irradiated and irradiated groups. Data are presented as mean ± SD from three independent biological experiments.

In non-irradiated cells, intracellular ROS levels showed only minor variations across the tested GB concentrations (Figure 2A). Likewise, the percentage of live cells remained comparable among all treatment groups (Figure 2B). No consistent concentration-dependent changes in either ROS generation or live-cell percentage were observed following GB pretreatment alone.

A similar pattern was observed after exposure to 3 Gy irradiation. Neither intracellular ROS levels nor the percentage of live cells differed consistently between irradiated and non-irradiated cells at the corresponding GB concentrations. Visual examination of the data revealed no reproducible effects of irradiation on intracellular ROS generation or the percentage of live cells (Figures 2B, C).

To further evaluate these observations, a two-way analysis of variance (ANOVA) was performed using GB concentration and irradiation as independent factors. For intracellular ROS levels, the analysis revealed no significant main effects of GB concentration (*F* (4, 20) = 0.095, *p* = 0.983), no significant main effects of irradiation (*F* (1, 20) = 0.261, *p* = 0.615), and no significant interaction between GB concentration and irradiation (*F* (4, 20) = 0.491, *p* = 0.743). Similarly, analysis of the percentage of live cells demonstrated no significant effects of GB concentration (*F* (4, 20) = 0.233, *p* = 0.917), no significant effects of irradiation (*F* (1, 20) = 0.008, *p* = 0.931), and no significant GB × irradiation interaction (*F* (4, 20) = 0.098, *p* = 0.982).

Overall, these findings indicate that, under the present experimental conditions, 3 Gy irradiation did not significantly alter intracellular ROS levels or the percentage of live cells in MDA-MB-231 cells. Furthermore, GB pretreatment neither significantly affected these parameters nor modified the cellular response to irradiation. Thus, no evidence of a radiomodifying effects of GB were detected based on intracellular ROS generation or live-cell analysis.

### 3.3 Live cells analysis by apoptosis assay (FACS)

Live-cell percentages were determined based on Annexin V^−^/PI^−^ staining, independently of the live-cell analysis performed during the ROS assay. To evaluate the potential radiomodifying effects of *Ginkgo biloba* (GB), the percentage of live MDA-MB-231 cells was determined by flow cytometry following treatment with selected GB concentrations prior to exposure to 3 Gy irradiation. Live-cell populations were analyzed at 24, 48 and 72 h after treatment.

In the non-irradiated group, the percentage of live cells remained comparable across all GB concentrations at each observation time (Figure 3). Although minor variations were observed between individual treatment groups, no consistent concentration or time dependent pattern was evident.

**Figure 3:**
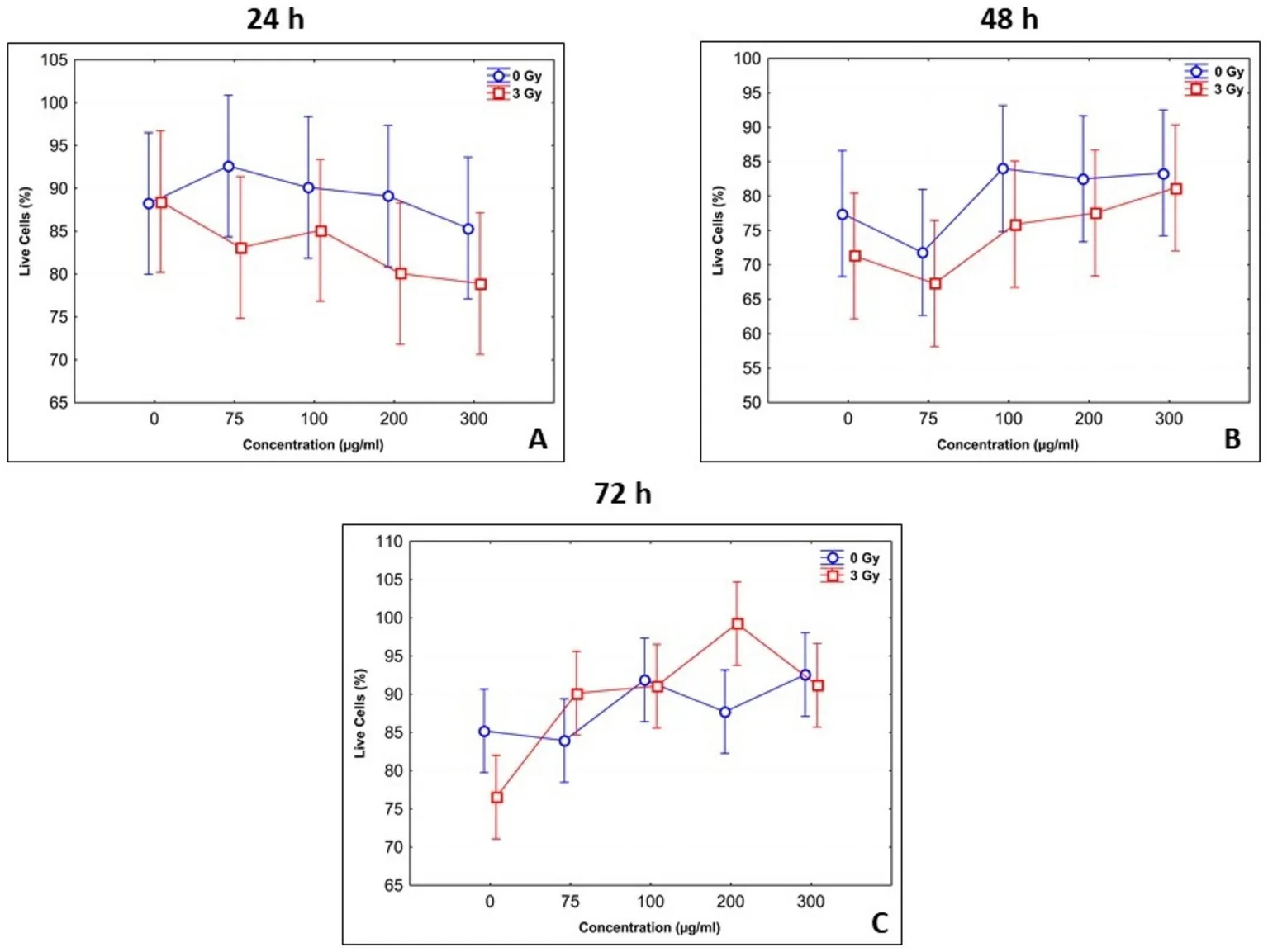
Effects of *Ginkgo biloba* (GB) treatment on the percentage of live MDA-MB-231 cells assessed by apoptosis assay using flow cytometry following exposure to 3 Gy irradiation. Cells were treated with increasing concentrations of GB (0–300 μg/mL), and viable cell populations were analyzed at 24 h **(A)**, 48 h **(B)**, and 72 h **(C)** hours in both non-irradiated and irradiated groups. Data are presented as mean ± SD from three independent biological experiments.

Similarly, irradiation with 3 Gy did not produce a consistent reduction in the percentage of live cells compared with the corresponding non-irradiated groups. Visual examination of the data did not indicate a reproducible effect of irradiation on cell survival at any GB concentration or time point. The percentages of early apoptotic and late apoptotic/necrotic (LAN) cells are presented in Supplementary Figures S1 and S2.

To further evaluate these observations, a two-way analysis of variance (ANOVA) was performed separately for each time point using GB concentration and irradiation as independent factors. GB concentration did not significantly affect the percentage of live cells (24 h: *F* (4, 20) = 0.208, *p* = 0.931; 48 h: *F* (4, 20) = 0.642, *p* = 0.639; 72 h: *F* (4, 20) = 1.747, *p* = 0.179). Likewise, irradiation alone had no significant effects (24 h: *F* (1, 20) = 1.298, *p* = 0.268; 48 h: *F* (1, 20) = 0.799, *p* = 0.382; 72 h: *F* (1, 20) = 0.158, *p* = 0.695). Furthermore, no significant interaction between GB concentration and irradiation was detected (24 h: *F* (4, 20) = 0.113, *p* = 0.977; 48 h: *F* (4, 20) = 0.028, *p* = 0.998; 72 h: *F* (4, 20) = 1.004, *p* = 0.429).

Overall, these findings indicate that neither GB pretreatment nor 3 Gy irradiation significantly affected the percentage of live MDA-MB-231 cells under the present experimental conditions. Furthermore, GB did not significantly modify the cellular response to irradiation, indicating the absence of a radiomodifying effects under the present experimental conditions.

## 4 Discussion

The present study investigated whether *Ginkgo biloba* (GB) extract modifies the response of MDA-MB-231 triple-negative breast cancer (TNBC) cells to ionizing radiation. The principal finding was that GB exerted a concentration-dependent cytotoxic effect, whereas no statistically significant radiomodifying effects were detected using three independent biological endpoints: cell viability, intracellular reactive oxygen species (ROS) generation, and apoptosis. Two-way ANOVA further demonstrated that neither 3 Gy irradiation nor the GB × irradiation interaction significantly affected these parameters. Collectively, these findings indicate that GB exhibited intrinsic cytotoxic activity but did not modify the radiation response of MDA-MB-231 cells under the present experimental conditions.

The search for effective radiosensitizers remains an important objective in radiation oncology because selective enhancement of tumour radiosensitivity has the potential to improve therapeutic efficacy without increasing radiation injury to surrounding normal tissues (29). However, the clinical application of conventional radiosensitizers is frequently limited by systemic toxicity and adverse effects (29). Consequently, naturally occurring bioactive compounds have attracted increasing interest owing to their favourable safety profiles (30) and their ability to modulate multiple signalling pathways involved in oxidative stress, inflammation, apoptosis and cellular metabolism (31, 32).

Among these compounds, GB has received considerable attention because its flavonoids and terpene lactones regulate oxidative stress, mitochondrial function and cell survival signalling (19, 33). Previous studies have demonstrated antiproliferative and pro-apoptotic effects of GB in several tumour models, including breast cancer (34–37). These findings are consistent with our observation that increasing GB concentrations reduced the viability of MDA-MB-231 cells. However, the primary objective of the present study was not to evaluate the intrinsic cytotoxicity of GB but to determine whether this cytotoxic activity translated into enhanced cellular responses to ionizing radiation. Our results did not support this hypothesis. Although higher GB concentrations reduced cell viability, two-way ANOVA identified GB concentration as the only significant determinant of viability, whereas neither irradiation nor the GB × irradiation interaction reached statistical significance. Thus, the reduction in viability was attributable to the intrinsic cytotoxic effects of GB rather than to radiosensitization.

An important consideration when interpreting these findings is the experimental design. A single dose of 3 Gy was selected because it represents a clinically relevant dose per fraction used in modern radiotherapy while permitting detection of potential radiomodifying effects without excessive radiation-induced cytotoxicity that might obscure subtle treatment interactions. However, under the present experimental conditions, 3 Gy irradiation alone did not produce a statistically significant effects on cell viability, intracellular ROS generation, or the proportion of live cells. This represents a limitation of the present study because a limited response to irradiation alone reduces the experimental dynamic range available for detecting a potential GB × irradiation interaction. Therefore, the absence of a statistically significant interaction should not be interpreted as definitive evidence that GB lacks radiosensitizing activity, but rather as an indication that no radiomodifying effects were detectable at 3 Gy using the investigated endpoints and experimental conditions.

Another important factor is the intrinsic radio-resistance of MDA-MB-231 cells. This cell line is characterized by efficient DNA damage responses, activation of prosurvival signalling pathways and high adaptive capacity, resulting in relatively low sensitivity to clinically relevant radiation doses. The limited response to 3 Gy may, at least in part, reflect the intrinsic radioresistant phenotype of MDA-MB-231 cells; however, this biological characteristic does not eliminate the limitation associated with using a single radiation dose. These biological characteristics may reduce the likelihood of detecting modest radiosensitizing effects. Accordingly, the absence of a significant GB × irradiation interaction is biologically plausible and consistent with the known radioresistant phenotype of MDA-MB-231 cells.

The choice of biological endpoint is an important consideration when interpreting the present findings. The MTT assay was employed to evaluate short-term changes in cellular metabolic viability following GB treatment and irradiation and was complemented by measurements of intracellular ROS and Annexin V/PI-defined cell populations to assess oxidative stress and cell-death-associated responses. However, MTT primarily reflects cellular metabolic activity and does not directly determine the long-term reproductive capacity of irradiated cells. This distinction is particularly relevant in radiobiology, as cells may retain metabolic activity for a period following irradiation despite sustaining damage that ultimately prevents continued proliferation and colony formation. Reactive oxygen species (ROS) play a central role in radiation-induced cellular injury by contributing to DNA damage, oxidative stress and activation of cell death pathways (38). Flavonoids present in standardized GB extracts, including EGb 761, have been reported to modulate oxidative stress through ROS scavenging and regulation of antioxidant defence systems (39, 40). However, intracellular ROS measurements in the present study revealed no significant effects of GB pretreatment, irradiation or their interaction. Moreover, irradiation alone did not significantly increase ROS levels, suggesting that oxidative stress was efficiently controlled in this experimental model. Together with the known radioresistant phenotype of MDA-MB-231 cells, these findings indicate that modulation of intracellular ROS is unlikely to account for the concentration-dependent cytotoxicity of GB or to represent a mechanism of radio-modification under the present experimental conditions.

Flow cytometric analysis provided independent confirmation of the MTT and ROS findings. Neither GB pretreatment nor irradiation significantly affected the proportion of viable cells, and no significant GB × irradiation interaction was detected at any time point. Although GB reduced metabolic activity in the MTT assay, these effects were not accompanied by a corresponding decrease in the percentage of viable cells measured by Annexin V/PI staining. This apparent discrepancy most likely reflects the different biological endpoints assessed by the two methods: whereas the MTT assay primarily measures mitochondrial metabolic activity, Annexin V/PI staining evaluates membrane integrity and phosphatidylserine externalization associated with apoptosis. Consequently, reduced metabolic activity does not necessarily indicate immediate apoptotic cell death. Moreover, the reduction in MTT-derived metabolic viability in the absence of a corresponding decrease in Annexin V/PI-defined viable cells may also be compatible with a cytostatic response, in which GB suppresses cellular proliferation or induces cell-cycle arrest without causing immediate apoptotic cell death. However, because cell-cycle distribution and proliferation were not directly evaluated in the present study, this interpretation remains speculative. Future studies incorporating cell-cycle analysis and proliferation-based assays will be required to distinguish cytostatic effects from alterations in cellular metabolism or delayed cell death (41, 42).

Previous studies have shown that GB may regulate apoptosis through modulation of Bax/Bcl-2 expression, activation of caspase-3 and mitochondrial signalling pathways (43). Although these mechanisms probably contribute to the intrinsic cytotoxic activity of GB reported in several tumour models, they did not translate into enhanced radiation-induced apoptosis in the present study. Together with the MTT and ROS results, these findings consistently indicate that GB affected tumour cell metabolism without producing measurable radiomodification under the present experimental conditions.

Several studies have reported radiosensitizing effects of GB or its constituents under specific experimental conditions. For example, Kim et al. demonstrated enhanced radiation responses in a C3H mouse fibrosarcoma model without increasing acute normal tissue injury, an effect attributed primarily to improved tumour perfusion and reduced hypoxia rather than to direct cellular radiosensitization (44–46). Differences in tumour model, experimental system, microenvironment and biological endpoints may therefore explain the apparent discrepancy between those observations and the present results. Unlike the *in vivo* model investigated by Kim et al., our study employed a single radioresistant TNBC cell line cultured under normoxic *in vitro* conditions, where hypoxia-dependent mechanisms are unlikely to contribute substantially to radiation response. Consequently, the absence of a detectable radiomodifying effects in the present study does not necessarily contradict previous reports but rather suggests that any potential radiomodifying activity of GB is likely to be context-dependent.

From a translational perspective, these findings should be interpreted cautiously. The present *in vitro* data do not provide evidence that GB enhances the radiation response of MDA-MB-231 cells under the conditions tested, but they also do not establish that GB is clinically neutral or safe during radiotherapy. Because only one tumour cell line, one radiation dose, and short-term cellular endpoints were examined, no direct recommendation regarding the use of GB by patients undergoing radiotherapy can be made from the present study.

Future studies should therefore include additional breast cancer models with different intrinsic radiosensitivities, clonogenic survival assays, fractionated irradiation protocols and molecular analyses of DNA damage response and oxidative stress pathways. Such investigations will clarify whether GB may exhibit radiomodifying activity under alternative experimental conditions.

## 5 Conclusion

The present study demonstrates that *Ginkgo biloba* (GB) extract exerts a concentration-dependent cytotoxic effects on MDA-MB-231 triple-negative breast cancer cells, as shown by the MTT assay. However, GB pretreatment had no significant effects on intracellular reactive oxygen species (ROS) generation or the percentage of live cells assessed by flow cytometry. Furthermore, 3 Gy irradiation did not significantly affect these endpoints, and no significant interaction between GB pretreatment and irradiation was detected in any of the experimental assays. Therefore, under the present experimental conditions, no statistically significant radiomodifying effects of GB were detectable following a single 3 Gy exposure using the investigated short-term cellular endpoints.

These findings indicate that, although GB exhibits intrinsic cytotoxic activity at higher concentrations, it does not significantly alter the response of MDA-MB-231 cells to ionizing radiation. Further studies using additional breast cancer models, different radiation doses, clonogenic survival assays, and mechanistic molecular analyses are warranted to determine whether GB may influence radiation responses under other experimental conditions.

## 6 Limitations of the study

Several limitations should be considered when interpreting these findings. First, only one radioresistant TNBC cell line was investigated. Second, the biological response was evaluated following a single clinically relevant radiation dose, consequently, the effects of multiple radiation doses or fractionated radiation regimens remain to be characterized. Third, while a fixed concentration of GB was utilized based on prior screening, evaluating a broader range of different concentrations of GB could provide deeper insights into dose-dependent interactions. Fourth, clonogenic survival, which remains the reference endpoint for assessment of radiosensitivity, was not included. In addition, the use of three independent biological replicates per experimental condition limits statistical power, particularly for detecting modest treatment and interaction effects, and reduces the precision with which distributional assumptions can be evaluated; therefore, the absence of statistically significant effects should be interpreted cautiously. Finally, the present study focused on functional cellular endpoints and did not investigate molecular pathways involved in DNA damage signalling, cell-cycle regulation or DNA repair.

## Data Availability

The original contributions presented in the study are included in the article/Supplementary material, further inquiries can be directed to the corresponding author.
