# Supplementary material for: Evaluation of the radiomodifying effects of Ginkgo biloba in radioresistant triple negative breast cancer cells
Source: Front Public Health. 2026 Oct 5;14:1952136. doi: 10.3389/fpubh.2026.1952136 (PMC13677538; doi:10.3389/fpubh.2026.1952136)
Supplement: Supplementary file 1 [file Supplementary_file_1.docx]

**Figure S1.** Effect of Ginkgo biloba (GB) treatment on the percentage of early apoptotic MDA-MB-231 cells assessed by apoptosis assay using flow cytometry following exposure to 3 Gy irradiation. Cells were treated with increasing concentrations of GB (0–300 µg/ml), and the percentage of early apoptotic populations were determined at 24 h (A), 48 h (B), and 72 h (C) hours in both non-irradiated and irradiated groups. Data are presented as mean ± SD from three independent biological experiments.

**Figure S2.** Effect of Ginkgo biloba (GB) treatment on the percentage of Late apoptotic/necrotic (LAN) MDA-MB-231 cells assessed by apoptosis assay using flow cytometry following exposure to 3 Gy irradiation. Cells were treated with increasing concentrations of GB (0–300 µg/ml), and the percentage of LAN cell populations were determined at 24 h (A), 48 h (B), and 72 h (C) hours in both non-irradiated and irradiated groups. Data are presented as mean ± SD from three independent biological experiments.
